# Goals of Care Among Patients With Advanced Cancer and Their Family Caregivers in the Last Years of Life

**DOI:** 10.1001/jamanetworkopen.2024.5866

**Published:** 2024-04-11

**Authors:** Semra Ozdemir, Isha Chaudhry, Chetna Malhotra, Irene Teo, Eric Andrew Finkelstein

**Affiliations:** 1 Lien Centre for Palliative Care, Signature Programme in Health Services and System Research, Duke-NUS Medical School, Singapore; 2 Department of Population Health Sciences, Duke Clinical Research Institute, Duke University, Durham, North Carolina; 3 Department of Psychosocial Oncology, National Cancer Centre, Singapore; 4 Saw Swee Hock School of Public Health, National University of Singapore, Singapore

## Abstract

**Importance:**

Understanding goals of care for terminally ill patients at the end of life is crucial to ensure that patients receive care consistent with their preferences.

**Objectives:**

To investigate the patterns of goals of care among patient-caregiver dyads over the last years of the patient’s life and the associations of the goals of care with patient-caregiver characteristics.

**Design, Setting, and Participants:**

This prospective cohort study of 210 patient-caregiver dyads involved surveys every 3 months from July 8, 2016, until the patient’s death or February 28, 2022. Data from the last 2 years of the patients’ lives were analyzed. Dyads, which comprised patients with stage IV solid cancer and their caregivers, were recruited from outpatient clinics at 2 major cancer centers in Singapore.

**Main Outcomes and Measures:**

Goals of care were examined via the tradeoffs between life extension and symptom management and between life extension and cost containment. The actor-partner interdependence framework was implemented using mixed-effects linear regressions.

**Results:**

This study included 210 dyads (patients: mean [SD] age, 62.6 [10.5] years; 108 men [51.4%]; caregivers: mean [SD] age, 49.4 [14.6] years; 132 women [62.9%]). On average, 34% of patients (264 of 780 observations; range, 23%-42%) and 29% of caregivers (225 of 780 observations; range, 20%-43%) prioritized symptom management over life extension, whereas 24% of patients (190 of 780 observations; range, 18%-32%) and 19% of caregivers (148 of 780 observations; range, 8%-26%) prioritized life extension. Between cost containment and life extension, on average, 28% of patients (220 of 777 observations; range, 22%-38%) and 17% of caregivers (137 of 780 observations; range, 10%-25%) prioritized cost containment, whereas 26% of patients (199 of 777 observations; range, 18%-34%) and 35% of caregivers (271 of 780 observations; range, 25%-45%) prioritized life extension. Goals of care did not change as patients approached death. Patients prioritized symptom management if they experienced higher symptom burden (average marginal effect [SE], 0.04 [0.01]), worse spiritual well-being (average marginal effect [SE], −0.04 [0.01]), and accurate (vs inaccurate) prognostic awareness (average marginal effect [SE], 0.40 [0.18]) and if their caregivers reported accurate prognostic awareness (average marginal effect [SE], 0.53 [0.18]), lower impact of caregiving on finances (average marginal effect [SE], −0.28 [0.08]), and poorer caregiving self-esteem (average marginal effect [SE], −0.48 [0.16]). Compared with patients, caregivers expressed lower preferences for cost containment (average marginal effect [SE], −0.63 [0.09]). Patients prioritized cost containment if they were older (average marginal effect [SE], 0.03 [0.01]), had higher symptom burden (average marginal effect [SE], 0.04 [0.01]), had poorer spiritual well-being (average marginal effect [SE], −0.04 [0.01]), and their caregivers reported poorer caregiving self-esteem (average marginal effect [SE], −0.51 [0.16]) and more family support (average marginal effect [SE], −0.30 [0.14]).

**Conclusions and Relevance:**

In this cohort study of patient-caregiver dyads, findings suggested the importance of interventions aimed at reducing discordance in goals of care between patients and caregivers and helping them develop realistic expectations to avoid costly, futile treatments.

## Key Points

**Question:**

What are the patterns of goals of care among patients with advanced cancer and their family caregivers over the last 2 years of a patient’s life?

**Findings:**

In this cohort study of 210 patient-caregiver dyads, patients (28%) exhibited a stronger preference toward cost containment compared with their caregivers (17%). There were no significant changes in the goals of care between patients and caregivers as patients approached death.

**Meaning:**

The study findings suggest that interventions that target prognostic awareness and aim to reduce discordance in goals of care between patients and caregivers can assist in developing realistic care expectations, ultimately preventing the use of costly and ineffective treatments.

## Teaser

This cohort study investigates the patterns of goals of care among patients with advanced cancer and their family caregivers over the last years of the patient’s life and the associations of goals with patient and caregiver characteristics.

## Introduction

Patients with cancer experience reduced quality of life that worsens as their illness progresses.^1,2^ Although life-extending treatments can be effective in the earlier stages of the disease, their benefits become increasingly unclear as the illness advances. Consequently, patients with advanced cancer often face difficult decisions for which they weigh the potential benefits of life-extending treatments against the additional pain, symptoms, and higher costs.^3,4,5^ Previous research has focused primarily on the tradeoffs between life extension and symptom management, revealing generally stable goals over time.^6,7,8,9^

In this decision-making process, family caregivers often play integral roles.^10^ However, caregivers often have different treatment goals than patients,^11,12^ with some cross-sectional evidence indicating that caregivers typically prioritize life extension, while patients prioritize symptom management.^13,14,15,16^ This discrepancy is concerning because it can result in patients receiving care incongruent with their own goals, particularly if they lack decision-making capacity.^12^ Despite this risk, few studies have investigated the evolution of caregivers’ goals of care and how they differ from patients’ goals. Patient and caregiver goals of care are influenced by their characteristics.^17^ Previous studies have shown that patients who were married,^18,19^ experienced unplanned hospitalizations,^20,21^ and faced higher financial difficulties^22^ tended to prioritize life extension. Conversely, older patients^4,23^ and those with a correct understanding of their prognosis,^24,25,26^ higher symptom burden,^4,23^ and greater spiritual well-being^18,27^ were more inclined toward prioritizing symptom management. In addition, the goals of care were life prolongation if caregivers reported a lower caregiving burden,^28^ higher financial difficulties,^29^ and higher caregiving self-esteem.^11,28^ Social and family support also emerge as key determinants of care preferences, measured by lack of family support to caregivers, patient-caregiver coresidence, and the quality of relationships between patients and caregivers. Provision of unpaid care to others is also part of this category because most patients wish to avoid becoming burdens to their families.^30,31^ The nature of the patient-caregiver relationship, whether the caregiver is a spouse or an adult child, may further influence family support dynamics.^32,33,34^

Existing studies have primarily explored independent associations between patient and caregiver goals. However, these associations are likely interdependent, as suggested by the actor-partner framework, indicating that each dyad member’s characteristics may influence both themselves (ie, actor) and their partner’s outcomes.^35^ This framework has been used to understand interdependent relationships, including decision-making role preferences^36^ and health care experiences among patients and their caregivers.^37^

**Figure 1.**
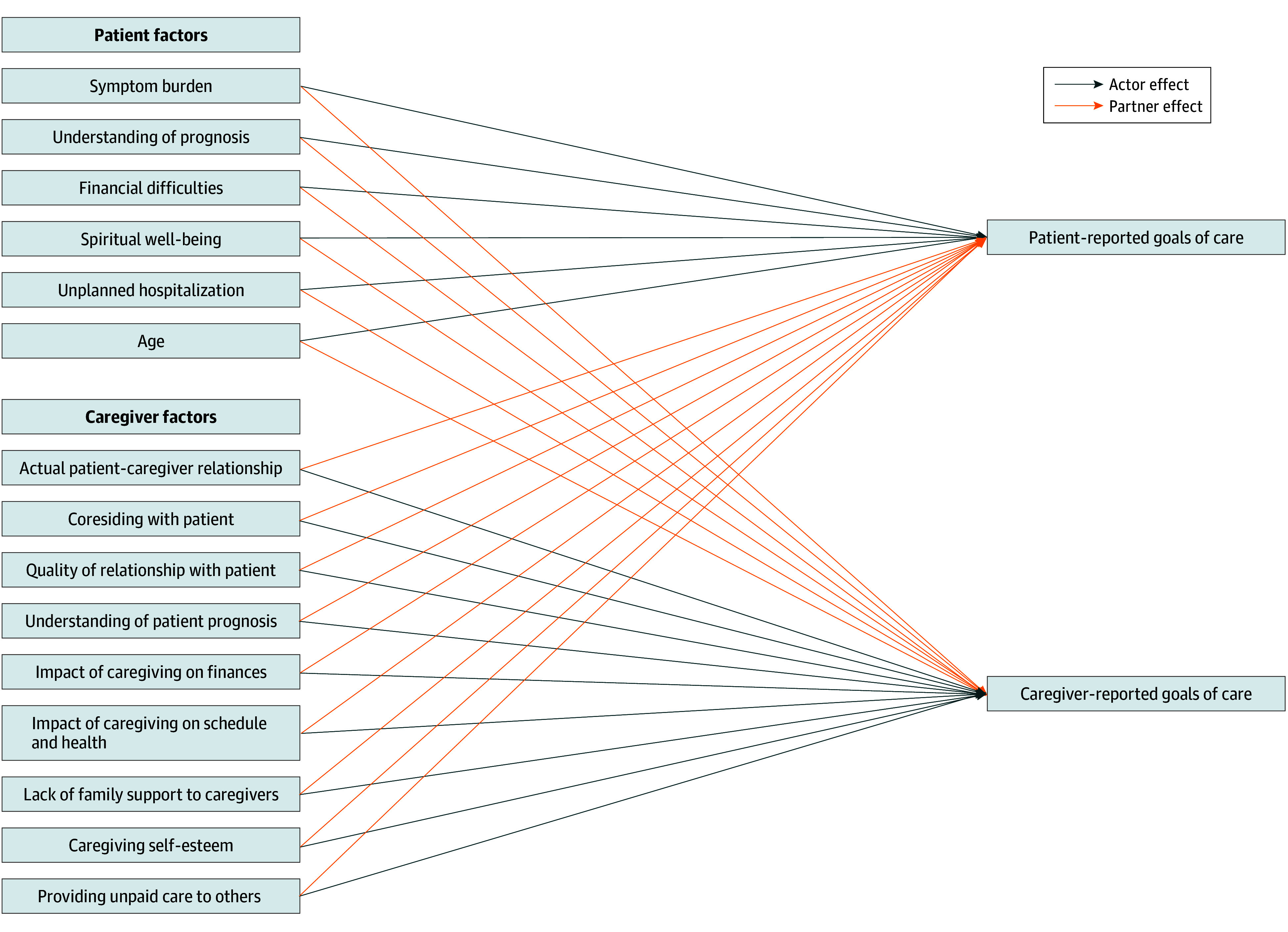
Conceptual Model: Patient-Caregiver Goals of Care Using Actor-Partner Interdependence Framework

The first objective of this study was to examine the patterns of patient and caregiver goals of care in the last 2 years of life, focusing on life extension vs symptom management and life extension vs treatment cost containment. The second objective was to investigate the associations of these goals with patient and caregiver characteristics, using the actor-partner interdependence framework (Figure 1).

## Methods

### Study Setting and Participants

Data for this study were obtained from the COMPASS (Cost of Medical Care of Patients With Advanced Serious Illness in Singapore) study,^38^ a prospective cohort study involving patients with stage IV solid cancer and their caregivers (NCT02850640). The eligibility criteria for patients were receiving a diagnosis of stage IV solid cancer, being 21 years of age or older, being a Singaporean citizen or permanent resident, being cognitively able to consent, and having an Eastern Cooperative Oncology Group^39^ performance status of 2 or less. Caregivers were considered eligible if they were the primary individual responsible for providing care or ensuring the provision of care or were involved in making treatment decisions on behalf of the patients. Participants were recruited between July 8, 2016, and March 5, 2018, from the medical oncology departments of 2 major public hospitals. Study participants were administered the baseline questionnaire and surveyed quarterly until patient death. The present study used data from the last 2 years of life for patients who died between November 14, 2016, and February 28, 2022, along with data from their caregivers. The study was approved by the SingHealth Centralised institutional review board. Written informed consent was provided by all participants. More information about the study can be found in the study protocol.^38^ This article followed the Strengthening the Reporting of Observational Studies in Epidemiology (STROBE) reporting guideline checklist for cohort studies.

### Outcomes

Goals of care questions were adapted from the CanCORS Study.^40,41^ Dyads were asked: “If you had to make/recommend a choice now, would you prefer/recommend treatment that extends life as much as possible, or would you want/recommend a treatment that gives minimal pain and discomfort (or: that costs less) (for the patient)?” Participants responded on a scale of 1 to 9, where 1 represented maximal life extension with severe pain or discomfort or with higher costs, 5 represented moderate life extension and moderate pain or discomfort or cost, and 9 indicated no life extension but minimal pain or discomfort or lower costs. Scores 1 to 4 were categorized as prioritizing life extension, and scores 6 to 9 were categorized as prioritizing symptom management or cost containment. Prior work has shown this measure to be associated with patient health care spending and use, indicating convergent validity for this measure.^42^

### Independent Variables

#### Patient Symptom Burden

The FACIT-Pal-14 (Functional Assessment of Chronic Illness Therapy–Palliative care) scale^43^ was used to identify the list of symptoms. Each symptom was rated on a Likert scale ranging from 0 to 4. Total scores ranged between 0 and 40, with higher scores indicating higher burden.

#### Patient Financial Difficulties

Patients were asked about the adequacy of their finances in (1) covering the cost of treatment, (2) meeting daily needs, and (3) affording small luxuries. A total score was calculated by summing the response options (range, 3-9; higher scores denoted greater financial difficulty).^44^

#### Patient Spiritual Well-Being

Spiritual well-being was assessed using the 12-item Functional Assessment of Chronic Illness Therapy–Spiritual Well-Being (FACIT-Sp) scale.^45^ Total scores ranged between 0 and 48. Higher scores indicated better spiritual well-being.

#### Patient and Caregiver Understanding of Patient Prognosis

Prognostic understanding was evaluated based on responses to the statement: “The current treatments you (/patient) are taking for your (/their) illness will cure you (/them).” Responses of no indicated prognostic awareness, while responses of yes and not sure indicated a lack of prognostic awareness.

#### Caregiving Burden and Esteem

Caregiving burden and self-esteem was assessed with the modified Caregiver Reaction Assessment Scale (CRA), which was validated for the Singaporean population.^46^ The CRA included 4 subscales measuring both negative and positive aspects of caregiving. The negative aspect subscales assessed the lack of family support, impact on finances, and impact on health and schedule, while the positive aspect subscale measured caregiving self-esteem. Higher scores on the caregiving self-esteem subscale indicated a greater positive association with caregiving, while higher scores on the other subscales indicated a greater negative association with caregiving.^46^

#### Quality of Patient-Caregiver Relationship

Caregiver-reported quality of relationship with the patient was assessed using a scale derived from the University of Southern California Longitudinal Study of Three-Generation Families.^47^ The total score ranges from 0 to 12, with a higher score indicating a better quality of relationship.

#### Providing Unpaid Care

Caregivers were asked whether they provided unpaid care to others while caring for the patient. Response options were yes and no.

#### Unplanned Admission

Patients’ hospitalization use was obtained from the medical records and linked to survey data to identify unplanned inpatient admissions (ie, through the accident and emergency facility) within the last 3 months of an assessment (yes or no).

#### Demographics

Patient age and sex were taken from medical records. Caregiver’s age, sex, relationship to the patient (spouse vs nonspouse), and coresidence (living together vs not) were obtained from the questionnaire.

### Statistical Analysis

We report the sample characteristics at baseline, which refers to the earliest assessment conducted within the last 2 years of the patient’s life. We present the distribution of goals of care for life extension vs (1) symptom management and (2) cost containment over this period.

To investigate the associations of patient-caregiver characteristics with their goals of care, we implemented the actor-partner interdependence framework using multivariable mixed-effects linear regressions (Figure 1). The dependent variables were the goals of care reported by the patients and caregivers, ranging from 1 to 9. The independent variables were the participant characteristics. We also included participant roles (patient [reference] vs caregiver) to investigate whether goals of care were different between patients and caregivers. The interaction terms between participant roles and participant characteristics were included to investigate how their own and each other’s characteristics may have influenced their goals of care. We included dyad identification numbers as a random effect and time from patient’s death as a linear fixed effect in all regressions. We estimated the average marginal effect (AME), which indicated the change in the estimated values of goals of care with a 1-unit change in each independent variable. We investigated whether the goals of care were significantly different between patients and caregivers by testing whether the AME for the caregiver variable was significant at the 95% confidence level. Similarly, we investigated whether the goals of care changed over time using the AME for the time from death variable. All covariates, except the actual patient-caregiver relationship, were time-varying variables.

We tested for the normality of residuals and assessed the variance inflation factor (<2.5) to assess multicollinearity after running the mixed-effects linear regressions. All *P* values were from 2-sided tests and results were deemed statistically significant at *P* < .05. We used Stata, version 17.1 (StataCorp LLC) for all analyses.

## Results

### Sample Characteristics

An initial 600 patients and 346 caregivers were enrolled in this study. Patients recruited without a caregiver (n = 289) and caregivers recruited without a patient (n = 35) were excluded from this study. Of the remaining 311 dyads, our sample included 210 dyads with a deceased patient (eFigure in Supplement 1). The median number of surveys answered by each participant was 3 (range, 1-9). The median (IQR) time to death for the patients who provided survey responses, measured in the last 2 years, was 9.5 months (IQR, 5.0-13.0 months), and the median time from the last survey assessment was 4.0 months (IQR, 2.0-6.0 months).

At baseline, the mean (SD) age of the patients was 62.6 (10.5) years, and the mean (SD) caregiver age was 49.4 (14.6) years (Table 1). A total of 102 patients (48.6%) and 132 caregivers (62.9%) were female. Half the caregivers were patients’ spouses (105 [50.0%]), and 162 of the caregivers (77.1%) resided with the patients. The mean (SD) patient symptom burden score was 7.2 (7.2) (range, 0-34), the spiritual well-being score was 37.2 (8.7) (range, 5-48), and the financial difficulties score was 6.2 (1.6) (range, 3-9). A total of 70 patients (33.3%) and 83 caregivers (39.5%) reported accurate prognostic awareness. Among patients, 30 (14.3%) experienced an unplanned hospitalization. The mean (SD) score for the caregiving impact on finances was 3.1 (1.2) (range, 1-5), the mean (SD) score for lack of family support was 2.2 (0.6) (range, 1-5), and the mean (SD) score for impact on schedule and health was 2.8 (0.8) (range, 1-5). The mean (SD) caregiving self-esteem score was 4.0 (0.6) (range, 1-5), and the mean (SD) quality of patient-caregiver relationship score was 9.0 (2.5) (range, 0-12). In addition, 97 of the caregivers (46.2%) provided unpaid care to others. We examined differences in baseline characteristics between those who completed all survey assessments (n = 13 dyads) and those who missed at least 1 assessment (n = 197 dyads) during the study period and found that the characteristics were not significantly different (eTable in Supplement 1).

**Table 1.** Sample Characteristics at Baseline

Characteristic	Patients (N = 210)	Caregivers (N = 210)
Age, mean (SD), y	62.6 (10.5)	49.4 (14.6)
Sex, No. (%)		
Female	102 (48.6)	132 (62.9)
Male	108 (51.4)	78 (37.1)
Patient characteristics		
Symptom burden (range, 0-34), mean (SD)	7.2 (7.2)	NA
Spiritual well-being (range, 5-48), mean (SD)	37.2 (8.7)	
Financial difficulties (range, 3-9), mean (SD)	6.2 (1.6)	
Understanding of prognosis, No. (%)		
Inaccurate	134 (63.8)	
Accurate	70 (33.3)	
Missing	6 (2.9)	
Any unplanned hospitalization within the last 3 mo of an assessment, No. (%)	30 (14.3)	
Caregiver characteristics		
Relationship with patient, No. (%)	NA	
Spouse		105 (50.0)
Adult child		83 (89.5)
Others		22 (10.5)
Coresiding with patient, No. (%)		162 (77.1)
Quality of patient-caregiver relationship score (range, 0-12), mean (SD)		9.0 (2.5)
Provides unpaid care to others, No. (%)		97 (46.2)
Understanding of prognosis, No. (%)		
Inaccurate		118 (56.2)
Accurate		83 (39.5)
Missing		9 (4.3)
Caregiver burden		
Impact on finances (range, 1-5), mean (SD)		3.1 (1.2)
Impact on schedule and health (range, 1-5), mean (SD)		2.8 (0.8)
Lack of family support (range, 1-5), mean (SD)		2.2 (0.6)
Caregiving self-esteem (range, 1-5), mean (SD)		4.0 (0.6)

Abbreviation: NA, not applicable.

### Patterns of Dyad Goals of Care in the Last 2 Years of Patient’s Life

#### Life Extension vs Symptom Management

**Figure 2.**
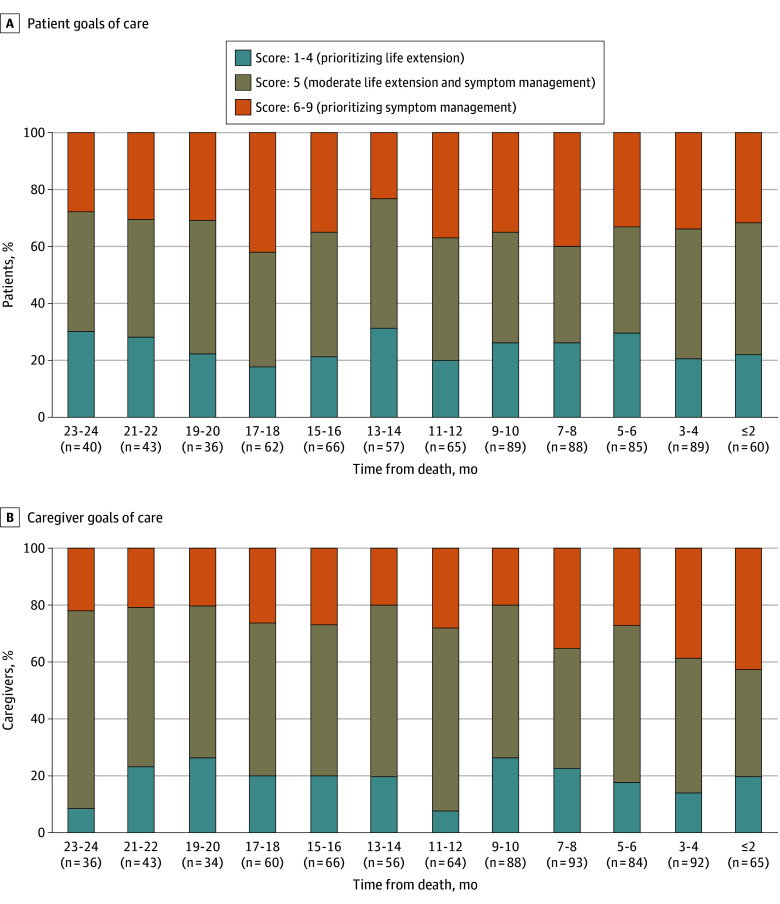
Patient-Caregiver Goals of Care in the Last 2 Years of Life: Life Extension vs Symptom Management

In the last 2 years of life, on average, 34% of patients (264 of 780 observations; range, 23%-42%) and 29% of caregivers (225 of 780 observations; range, 20%-43%) reported scores prioritizing symptom management (Figure 2). Conversely, on average, 24% of patients (190 of 780 observations; range, 18%-32%) and 19% of caregivers (148 of 780 observations; range, 8%-26%) reported scores prioritizing life extension. The most commonly stated goal was a balanced focus on life extension and symptom management, with 34% to 47% of patients and 37% to 69% of caregivers supporting this goal. These goals of care were not statistically different between patients and caregivers (AME [SE], −0.05 [0.10]; *P* = .63) (Table 2). Furthermore, they did not change over time for both patients (AME [SE], 0.01 [0.01]; *P* = .35) and caregivers (AME [SE], −0.02 [0.01]; *P* = .08).

**Table 2.** Mixed-Effects Linear Regression Estimates: AMEs for Associations Between Patient-Caregiver Goals of Care and Patient and Caregiver Characteristics

Characteristic	Goals of care: symptom management (vs life extension) (n = 202)				Goals of care: treatment-cost containment (vs life extension) (n = 202)			
	Patients		Caregivers		Patients		Caregivers	
	AME (SE)	*P* value	AME (SE)	*P* value	AME (SE)	*P* value	AME (SE)	*P* value
Role: caregiver (reference: patient)	NA		−0.05 (0.10)	.63	NA		-0.63 (0.09)	<.001
Time from death	0.01 (0.01)	.35	−0.02 (0.01)	.08	0.01 (0.01)	.31	−0.01 (0.01)	.26
Patient characteristics								
Age	0.02 (0.01)	.06	0.03 (0.01)	.001	0.03 (0.01)	.002	0.04 (0.01)	<.001
Symptom burden	0.04 (0.01)	<.001	0.03 (0.01)	.01	0.04 (0.01)	<.001	0.01 (0.01)	.24
Spiritual well-being	−0.04 (0.01)	<.001	−0.01 (0.01)	.20	−0.04 (0.01)	<.001	−0.003 (0.01)	.72
Financial difficulties	0.04 (0.05)	.46	−0.02 (0.05)	.69	0.10 (0.05)	.06	0.04 (0.05)	.44
Accurate understanding of prognosis	0.40 (0.18)	.03	−0.03 (0.18)	.86	0.22 (0.19)	.24	0.15 (0.19)	.41
Had an unplanned hospitalization within the last 3 mo of assessment	0.30 (0.21)	.16	−0.003 (0.21)	.99	0.41 (0.21)	.05	−0.25 (0.21)	.23
Caregiver characteristics								
Spouse (vs nonspouse)	−0.20 (0.23)	.38	−0.07 (0.23)	.76	−0.24 (0.24)	.32	0.39 (0.24)	.10
Coresiding with patient, yes (vs no)	−0.71 (0.26)	.006	−0.11 (0.25)	.68	0.44 (0.27)	.10	−0.19 (0.26)	.47
Quality of relationship with patient	0.06 (0.04)	.14	−0.07 (0.04)	.12	0.06 (0.04)	.18	−0.01 (0.04)	.75
Provides unpaid care to others	0.16 (0.16)	.34	0.17 (0.16)	.29	0.23 (0.16)	.16	0.63 (0.17)	<.001
Accurate understanding of prognosis	0.53 (0.18)	.003	0.28 (0.18)	.12	0.23 (0.18)	.22	0.25 (0.18)	.18
Impact of caregiving on finances	−0.28 (0.08)	.001	0.06 (0.08)	.47	0.13 (0.09)	.14	0.21 (0.09)	.02
Impact of caregiving on schedule and health	0.10 (0.13)	.41	0.02 (0.13)	.88	−0.07 (0.13)	.56	−0.13 (0.13)	.31
Lack of family support in caregiving	−0.04 (0.14)	.79	0.07 (0.13)	.59	−0.30 (0.14)	.03	0.03 (0.14)	.81
Caregiving self-esteem	−0.48 (0.16)	.002	−0.04 (0.16)	.82	−0.51 (0.16)	.001	−0.58 (0.16)	<.001

Abbreviations: AME, average marginal effect; NA, not applicable.

#### Life Extension vs Containing Treatment Costs

**Figure 3.**
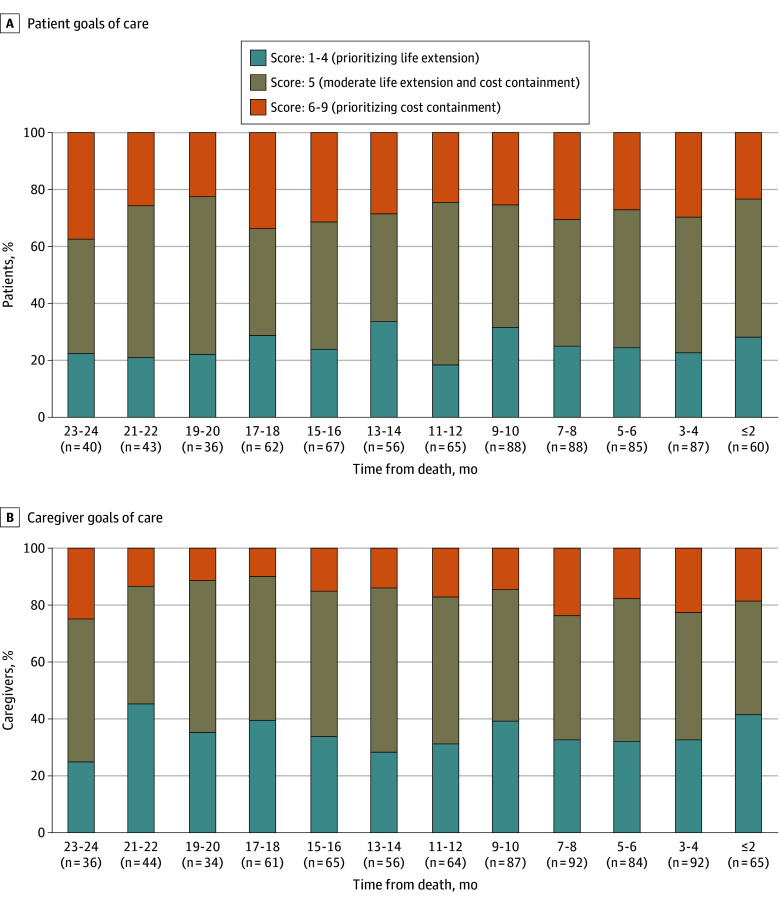
Patient-Caregiver Goals of Care in the Last 2 Years of Life: Life Extension vs Treatment-Cost Containment

On average, balancing between life extension and cost containment was the most common goal, reported by 37% to 57% of patients and 40% to 57% of caregivers. On average, 28% of patients (220 of 777 observations; range, 22%-38%) and 17% of caregivers (137 of 780 observations; range, 10%-25%) reported scores prioritizing treatment cost containment, while 26% of patients (199 of 777 observations; range, 18%-34%) and 35% of caregivers (271 of 780 observations; range, 25%-45%) reported scores prioritizing life extension (Figure 3). Compared with patients, caregivers reported a lower preference for cost containment (and a greater preference for life extension) (AME [SE], −0.63 [0.09]; *P* < .001). However, there were no significant changes over time in these goals for both patients (AME [SE], 0.01 [0.01]; *P* = .31) and caregivers (AME [SE], −0.01 [0.01]; *P* = .26) (Table 2).

### Factors Associated With Patient and Caregiver Goals of Care: An Actor-Partner Interdependence Framework

#### Life Extension vs Symptom Management

Patients with higher symptom burden (AME [SE], 0.04 [0.01]; *P* < .001), worse spiritual well-being (AME [SE], −0.04 [0.01]; *P* < .001) or accurate prognostic awareness (AME [SE], 0.40 [0.18]; *P* = .03) and those who did not reside with their caregivers (AME [SE], −0.71 [0.26]; *P* = .006) reported goals toward prioritization of symptom management over life extension (Table 2). This was also the case among patients whose caregivers reported accurate prognostic awareness (AME [SE], 0.53 [0.18]; *P* = .003), lower impact of caregiving on finances (AME [SE], −0.28 [0.08]; *P* = .001), and lower caregiving self-esteem (AME [SE], −0.48 [0.16]; *P* = .002). In addition, caregivers of older patients (AME [SE], 0.03 [0.01]; *P* = .001) and caregivers of patients with a higher symptom burden (AME [SE], 0.03 [0.01]; *P* = .01) also reported goals toward prioritizing symptom management (Table 2).

#### Life Extension vs Treatment Cost Containment

For patients, characteristics associated with a goal toward cost containment included being older (AME [SE], 0.03 [0.01]; *P* = .002), having a higher symptom burden (AME [SE], 0.04 [0.01]; *P* = .001), and having worse spiritual well-being (AME [SE], −0.04 [0.01]; *P* < .001) and caregivers reporting worse caregiving self-esteem (AME [SE], −0.51 [0.16]; *P* = .001) and lower impact of lack of family support in caregiving (AME [SE], −0.30 [0.14]; *P* = .03) (Table 2).

Among caregivers, prioritizing cost containment was also the goal for those with older patients (AME [SE], 0.04 [0.01]; *P* < .001), those who provided unpaid care to others (AME [SE], 0.63 [0.17]; *P* < .001), those who reported a higher impact of caregiving on their finances (AME [SE], 0.21 [0.09]; *P* = .02), and those who reported worse caregiving self-esteem (AME [SE], −0.58 [0.16]; *P* < .001) (Table 2). Patients’ financial difficulties, caregiver’s spousal relationship with the patient, the quality of the relationship, and the caregiving impact on schedule and health were not significantly associated with patient and caregiver goals of care.

## Discussion

This study examined the goals of care among patients and their caregivers regarding the tradeoffs between life extension and symptom management or cost containment during the last 2 years of a patient’s life. The results revealed that when faced with a choice, about half of the patient-caregiver dyads preferred a moderate approach, balancing life extension with symptom management or cost containment. These findings demonstrate that while considerations of quality of life and financial concerns remained salient, the goal of life extension still held significance for many dyads.

Supporting the findings of past research,^6,7,8,9^ the goals of care among patients and caregivers remained relatively stable as patients approached death. This finding raises concerns, as it suggests that many patients may hold unrealistic expectations regarding their care, potentially leading to the receipt of costly and futile life-extending treatments at the expense of a better quality of life toward the end of life.^48^ Future research incorporating qualitative interviews can provide insights into why some patients and caregivers did not prioritize symptom management even near death.

Contrary to past studies,^4^ there were no significant differences in the goals of care between patients and their caregivers regarding life extension vs symptom management. This is an important finding regarding end-of-life discussions within the family and for patients to receive care consistent with their goals. However, caregivers showed a lower preference than patients for cost containment, consistent with existing literature indicating that caregivers are generally willing to pay more than patients to improve patient’s health.^16,49^ This finding underscores patients’ conservative stance on spending on expensive treatments, indicating a potential area of focus for health care professionals and policymakers. Addressing this difference in goals of care can enhance communication and shared decision-making, ultimately improving outcomes.

Regression analyses demonstrated that when patients had lower quality of like, the goals of care focused toward prioritizing symptom management. This was evident among patients with higher symptom burden and worse spiritual well-being, as well as among caregivers of older patients and those with a greater symptom burden. Similarly, patients who reported lower quality of life were inclined to prioritize cost containment over life extension, including those with higher symptom burden and worse spiritual well-being. Furthermore, both older patients and their caregivers demonstrated a preference for containing treatment costs. Taken together, these findings suggest that when patients and caregivers recognize the extent of patient symptom burden, they are inclined to avoid costly life-extending treatments and prioritize symptom management.

Caregivers with lower caregiving self-esteem, indicating a lack of personal satisfaction or positive experiences from caregiving responsibilities,^50^ aligned with their patients in prioritizing cost containment. This finding suggests that their focus on cost containment instead of life extension may be influenced by their lack of fulfillment in the caregiving role. In addition, caregivers who reported a higher impact of caregiving on their finances and those who reported providing care to someone other than the patient also expressed a preference for prioritizing cost containment. These factors may be associated with their emphasis on cost containment as they consider the financial implications and responsibilities beyond the patient’s care.

The findings on participant characteristics have implications for tailoring end-of-life care to individual preferences. Health care professionals could prioritize comprehensive symptom management strategies over potentially life-extending treatments, particularly for patients with higher symptom burden and poor spiritual well-being. Ensuring accurate prognostic awareness among both patients and caregivers is pivotal to avoiding aggressive treatments. In addition, social and family support, such as quality of relationship and burden on caregivers, could serve as factors in identifying goals of care.

### Limitations

The findings should be interpreted within several limitations. First, the sample consisted of Singaporean patients with advanced cancer and their caregivers, which may limit the generalizability to other terminal illnesses, societies, or health care systems. Second, the survey questions did not state specific treatment options, and the measurement focused on general goals of care regarding life extension vs symptom management or cost containment. Third, the associations observed could be biased by the self-reporting behaviors of patients and caregivers.

## Conclusions

This cohort study found that when patients and caregivers were asked to make tradeoffs, a common goal of care among dyads was a moderate approach of balancing life extension against symptom management or cost containment. However, patients exhibited a stronger preference toward cost containment compared with their caregivers. This study also found no significant changes in the goals of care among patients and caregivers as patients approached death. These findings emphasize the need for interventions that reduce discordance in goals of care between patients and caregivers, helping them develop realistic care expectations and avoid costly, futile treatments.

## Notes

Supplement 1. **eTable.** Comparison of Baseline Characteristics: Dyads Who Completed All Survey Assessments (N=13) Versus Dyads Who Missed at Least One Assessment During the Study Period (N=197) **eFigure.** Flowchart of Study Participants

Supplement 2.
Nonauthor Collaborators

Supplement 3.
Data Sharing Statement
