# Supplementary material for: Goals of Care Among Patients With Advanced Cancer and Their Family Caregivers in the Last Years of Life
Source: JAMA Netw Open. 2024 Apr 11;7(4):e245866. doi: 10.1001/jamanetworkopen.2024.5866 (PMC11009823; doi:10.1001/jamanetworkopen.2024.5866)
Supplement: Supplement 1. — eTable. Comparison of Baseline Characteristics: Dyads Who Completed All Survey Assessments (N=13) Versus Dyads Who Missed at Least One Assessment During the Study Period (N=197) eFigure. Flowchart of Study Participants [file jamanetwopen-e245866-s001.pdf]

Supplementary Online Content

Ozdemir S, Chaudhry I, Malhotra C, Teo I, Finkelstein EA; Cost of Medical Care of Patients With Advanced Serious Illness in Singapore (COMPASS) Study Group. Goals of care among patients with advanced cancer and their family caregivers in the last years of life. *JAMA Netw Open.* 2024;7(4):e245866. doi:10.1001/jamanetworkopen.2024.5866

eTable. Comparison of Baseline Characteristics: Dyads Who Completed All Survey Assessments (N=13) Versus Dyads Who Missed at Least One Assessment During the Study Period (N=197)

eFigure. Flowchart of Study Participants

This supplementary material has been provided by the authors to give readers additional information about their work.

© 2024 Ozdemir S et al. *JAMA Network Open.*

eTable. Comparison of Baseline Characteristics: Dyads Who Completed All Survey Assessments (N=13) Versus Dyads Who Missed at Least One Assessment During the Study Period (N=197)

	**Patients**			**Caregivers**		
	Completers (n=13)	Non-completers (n=197)	p-value*	Completers (n=13)	Non-completers (n=197)	p-value*
Age, Mean (SD)	60.3 (12.5)	62.8 (10.4)	0.41	49.9 (14.3)	49.6 (14.6)	0.94
Gender, n(%)						
Female	8 (61.5)	94 (47.7)	0.33	8 (61.5)	124 (62.9)	0.92
Male	5 (38.5)	103 (52.3)		5 (38.5)	73 (37.1)	
**Patient characteristics**						
Symptom burden, range (0-34) Mean (SD)	8.6 (8.0)	7.1 (7.1)	0.46			
Spiritual well-being, range (5-48) Mean (SD)						
Financial difficulties, range (3-9) Mean (SD)	5.8 (1.4)	6.2 (1.6)	0.48			
Understanding of prognosis, n (%)	37.4 (6.9)	37.2 (8.8)	0.95			
Inaccurate	9 (69.2)	125 (63.4)	0.49			
Accurate	4 (30.8)	66 (33.5)				
Missing	0 (0)	6 (3.1)				
Any unplanned hospitalization within the last 3 months of an assessment, yes, n (%)	0 (0)	30 (15.2)	0.22			
**Caregiver characteristics**						
Relationship with patient, n(%)						
Spouse				6 (46.1)	99 (50.2)	0.77
Adult child				5 (38.5)	78 (39.6)	
Others				2 (15.4)	20 (10.1)	
Co-residing with patient, n(%)				12 (92.3)	150 (76.1)	0.31
Quality of patient-caregiver relationship, range (0-12), Mean (SD)						
Provides unpaid care to others, yes, n(%)				6 (46.2)	91 (46.2)	1.00
Understanding of prognosis, n (%)						
Inaccurate				6 (46.1)	112 (56.8)	0.43
Accurate				6 (46.1)	77 (39.1)	
Missing				1 (7.7)	8 (4.1)	
Caregiver burden						

© 2024 Ozdemir S et al. *JAMA Network Open.*

Impact on finance, range (1-5) Mean (SD)		3.6 (0.9)	3.0 (1.2)	0.10
Impact on schedule and health, range (1-5) Mean (SD)		3.1 (0.7)	2.8 (0.8)	0.24
Lack of family support, range (1-5) Mean (SD)		2.0 (0.3)	2.2 (0.6)	0.24
Caregiving self-esteem, range (1-5) Mean (SD)		4.0 (0.6)	4.0 (0.6)	0.89

*t-test was performed to compare statistical differences across groups for continuous measures and chi-square/ Fischer exact (if n<5) test were performed for categorical measures.

© 2024 Ozdemir S et al. *JAMA Network Open.*

eFigure. Flowchart of Study Participants

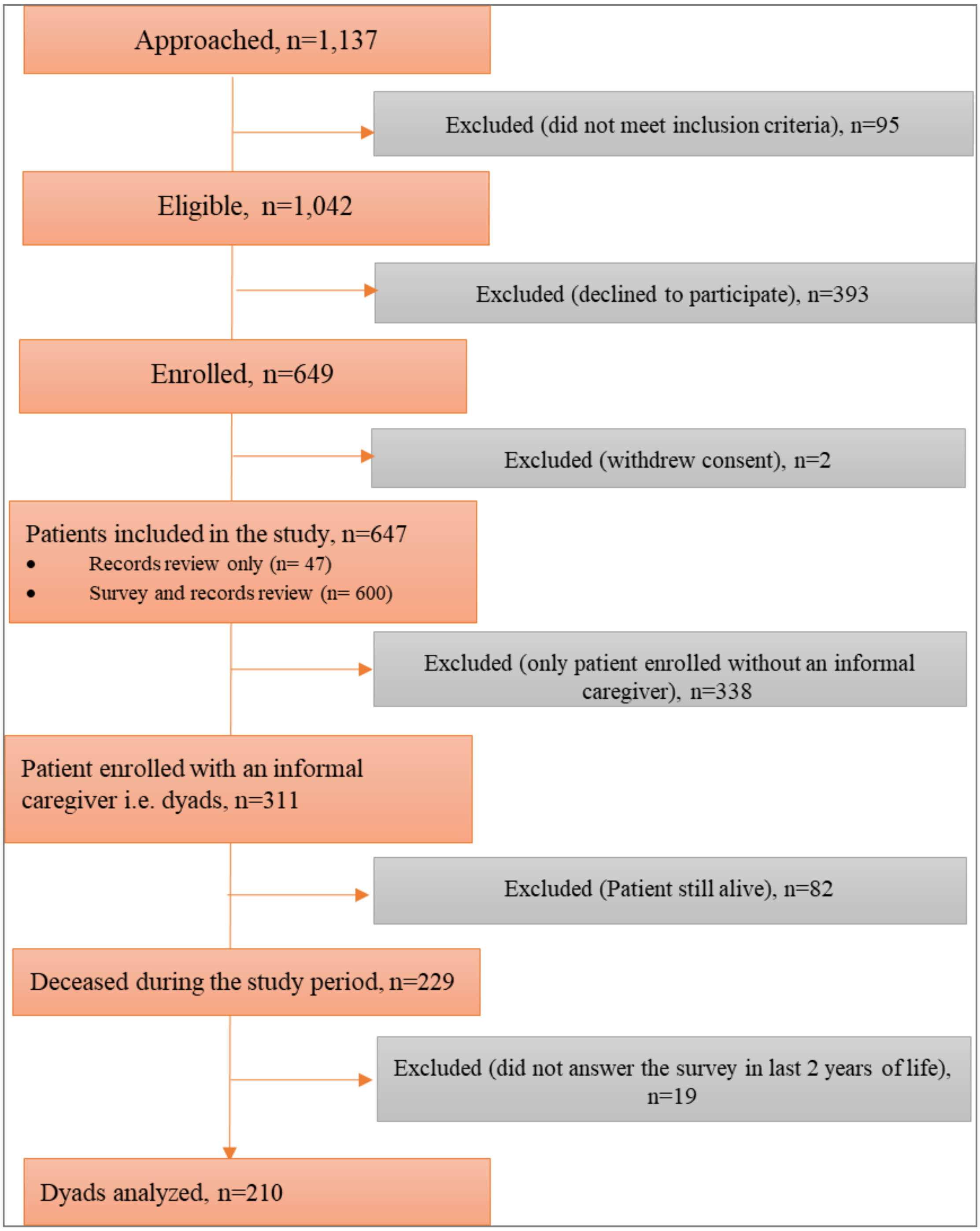

© 2024 Ozdemir S et al. *JAMA Network Open.*
