# Supplementary material for: Goals of Care Among Patients With Advanced Cancer and Their Family Caregivers in the Last Years of Life
Source: JAMA Netw Open. 2024 Apr 11;7(4):e245866. doi: 10.1001/jamanetworkopen.2024.5866 (PMC11009823; doi:10.1001/jamanetworkopen.2024.5866)
Supplement: Supplement 2. — Nonauthor Collaborators [file jamanetwopen-e245866-s002.pdf]

Supplemental Online Content: Nonauthor Collaborators
*First name, last name, and suffix (if applicable) are required and will appear in PubMed.

*Group Name(s): Cost of Medical Care of Patients with Advanced Serious Illness in Singapore (COMPASS) Study Group							
***First Name**	***Last Name**	***Suffix (eg, Jr, III)**	Academic Degrees	Institution	Location (city, state/province, country)	Role or Contribution, eg, chair, principal investigator	Group (if more than 1 Group listed in the byline) and/or Subgroup (eg, Steering Committee)
Ratna	Singh		MA	Duke-NUS Medical School	Singapore	Project Manager	
Rebecca A.	Dent		MD	National Cancer Centre Singapore	Singapore	Senior Consultant	
Yin Bun	Cheung		PhD	Duke-NUS Medical School	Singapore	Professor	
Rahul	Malhotra		MD	Duke-NUS Medical School	Singapore	Associate Professor	
Ravindran	Kanesvaran		MD	National Cancer Centre Singapore	Singapore	Senior Consultant	
Alethea Chung Pheng	Yee		MBBS	National Cancer Centre Singapore	Singapore	Senior Consultant	
Noreen	Chan		MBBS	National University Hospital	Singapore	Senior Consultant	
Huei Yaw	Wu		MBBS	Tan Tock Seng Hospital	Singapore	Senior Consultant	
Soh Mun	Chin		BS	Dover Park Hospice	Singapore	Director of Nursing	
Allyn Yin Mei	Hum		MBBCh	Tan Tock Seng Hospital	Singapore	Senior Consultant	
Grace Meijuan	Yang		MBBCh	National Cancer Centre Singapore	Singapore	Senior Consultant	
Patricia Soek Hui	Neo		MBBS	National Cancer Centre Singapore	Singapore	Senior Consultant	
Nivedita V	Nadkarni		PhD	Duke-NUS Medical School	Singapore	Assistant Professor	
