# Supplementary material for: Goals of Care Among Patients With Advanced Cancer and Their Family Caregivers in the Last Years of Life
Source: JAMA Netw Open. 2024 Apr 11;7(4):e245866. doi: 10.1001/jamanetworkopen.2024.5866 (PMC11009823; doi:10.1001/jamanetworkopen.2024.5866)
Supplement: Supplement 3. — Data Sharing Statement [file jamanetwopen-e245866-s003.pdf]

Data Sharing Statement

Ozdemir. Goals of Care Among Patients With Advanced Cancer and Their Family Caregivers in the Last Years of Life. *JAMA Netw Open*. Published April 11, 2024.
doi:10.1001/jamanetworkopen.2024.5866

Data
Data available: No

Additional Information
Explanation for why data not available: Data is available on reasonable request from the corresponding author.
